# Deep Frontal Lipoma With Frontal Bone Invasion: Report of a Rare Case

**DOI:** 10.7759/cureus.38546

**Published:** 2023-05-04

**Authors:** Yassine Ait M'barek, Lamia Benantar, Hajar Hamadi, Khalid Aniba

**Affiliations:** 1 Neurological Surgery, Ibn Tofail Hospital, Mohammed VI University Hospital, Marrakech, MAR

**Keywords:** lipoma, frontal lipoma, extra-dural hematoma, bone invasion, forehead lipoma

## Abstract

Lipomas are benign masses of fatty tissue, and in the forehead, they may develop in the subcutaneous or deep fat tissue. While subcutaneous lipomas are common, deep forehead lipomas are unusual and rarely invade the underlying bone. Only a few cases have been reported in the literature, and even fewer cases are reported in children. We present a case of a slowly growing frontal mass corresponding to a deep lipoma responsible for frontal bone invasion, resulting in a bony defect reaching the dural space. Through this case, we aim to emphasize forehead lipomas' clinical and surgical characteristics.

## Introduction

Lipomas are benign tumors of the fat tissue, typically presenting as asymptomatic subcutaneous nodules. Contrary to subcutaneous lipomas, deep-seated lipomas are solid and rigid with strong adherence to the surrounding tissues. They are usually misdiagnosed as other tumors -like sarcomas- due to their distribution between the fascial layers [1]. Information on lipomas developing in deep anatomical sites is scarce, particularly in children, which led us to present the following case and highlight its clinical, radiological, and surgical features.

## Case presentation

We present the case of an 8-year-old child born to nonconsanguineous parents with no significant medical history. She was admitted to our department - Department of Neurosurgery at Ibn Tofail Hospital, CHU Mohamed VI in Marrakech - with a spontaneous, slowly growing frontal mass discovered at birth. The mass was painless, progressively increasing in volume for the past 2 years. The patient reported no headaches, visual impairment, or vomiting.

**Figure 1:**
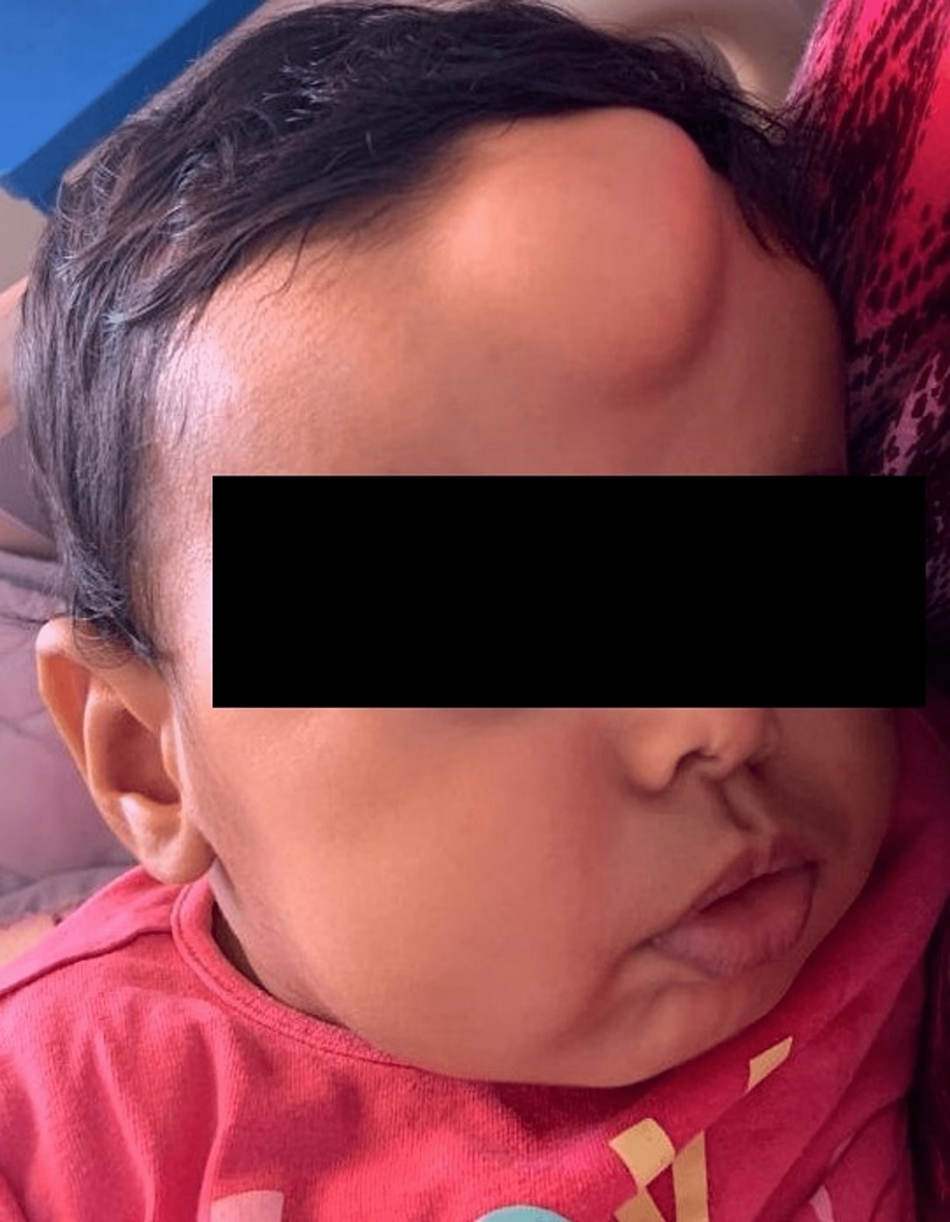
Preoperative image of the patient showing the frontal mass.

Clinical examination revealed a soft, painless, reductible frontal mass approximately 5 centimeters in diameter, with no signs of inflammation, covered by normal skin (Figure 1). The transillumination test was negative, and the visual assessment with fundoscopy was normal. Physical examination showed no other clinically apparent malformations. Differential diagnoses at this point were dermoid cysts, epidermoid cysts, hemangioma, and lymphangioma.

**Figure 2:**
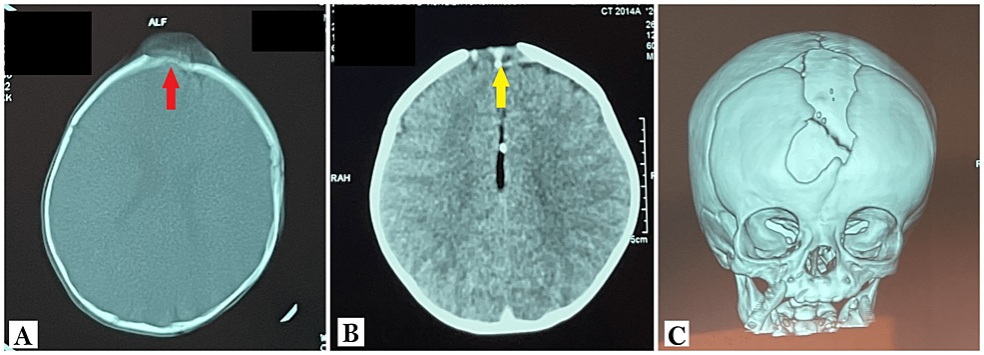
CT scan of the head showing the frontal mass. A: CT scan of the head in the bone window, in an axial plane, showing a well-defined isodense frontal mass (red arrow), with an invasion of the underlying bone resulting in a bony defect. B: CT scan of the head in the brain window, in an axial plane, showing the same lesion (yellow arrow) with no extension to the parenchyma and normal-looking lateral ventricles. C: CT scans 3D reconstruction showing the extent of the bony defect.

CT scan of the head revealed an iso-dense frontal mass measuring 5 cm in diameter, with sharp edges, responsible for a frontal bone defect. On the other hand, there was no associated hydrocephalus or parenchymal abnormalities (Figure 2).

The patient was programmed for surgical resection of the mass. A coronal incision was used to access the lesion. The skin incision for a large bilateral craniotomy -running from tragus to tragus- was performed, starting roughly 1 cm anteriorly to the main branch of the superficial temporal artery. The skin incision was performed carefully to spare the periosteum and remove the galea for a subsequent galeal flap. Dissection showed a hard frontal lesion, poorly vascularized, with a consistency similar to fat tissue. The lesion was invading the adjacent frontal bone, causing a large defect, irrupting the intracranial space, and coming in contact with the dura matter without invading it.

On the other hand, the lesion was not linked to the adjacent suture but was adherent to the deep fat tissue, requiring careful dissection. A parietal bone flap was used to reconstruct the frontal defect after dural suspension to prevent an extradural hematoma. Tight skin closure was performed with esthetic sutures. Immediate follow-up was uneventful. Forehead reconstruction was satisfying to the family and medical staff, and the scarring was minimal.

**Figure 3:**
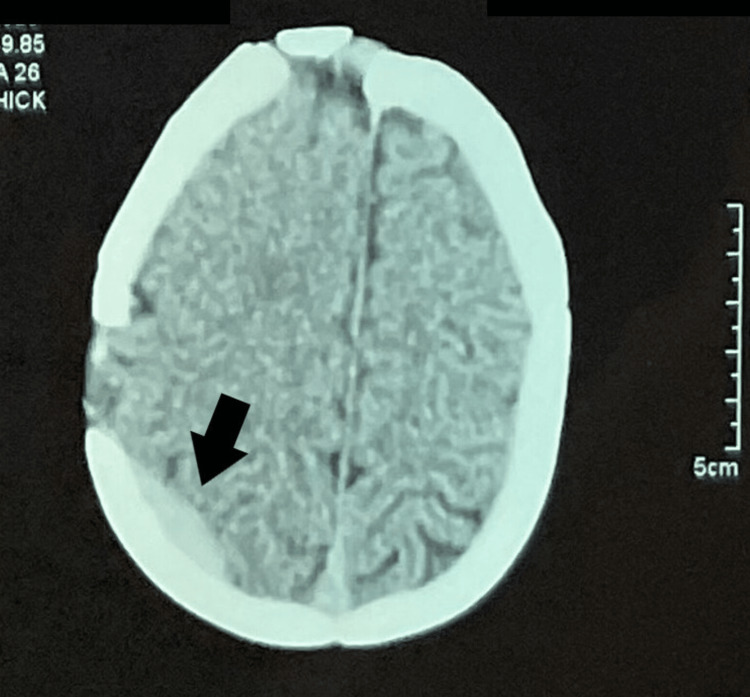
Control CT scan of the head showing a right parietal extradural hematoma (black arrow).

Two weeks later, the patient was admitted to the emergency department with headaches and vomiting. Glasgow Coma Scale on admission was 15/15, and neurological assessment was normal. CT scan of the head showed a right parietal extradural hematoma - probably due to the parietal bone flap used in the cranioplasty- measuring about 7x4mm with no mass effect. Additionally, a CT scan of the head also showed a satisfying removal of the mass with the frontal flap in place (Figure 3).

The patient was readmitted for observation and symptomatic treatment using analgesic and anti-emetic medications. During her hospital stay, she showed improvement in her initial complaints with full regression of the headaches and was discharged several days later. The anatomopathological assessment confirmed the suspected diagnosis of a lipoma and showed an erosive lipoma with minimal local necrosis, with no evidence of malignant progression.

## Discussion

Deep forehead lipoma was first described in late 1943 by Uriburu [2] and has since been confirmed in numerous reports as a separate clinicopathologic entity. Their prevalence is estimated to be 60% of all frontal lipomas, which can be related to the anatomical features of the forehead [3]. Although some instances suggest it may be connected to an inflammatory, endocrine, or traumatic processes, a clear etiology is still unknown [4,5]. They can develop inside the frontalis muscle, between the frontalis muscle and the deep investing fascia, between the deep fascia and the periosteum, or beneath the periosteum [2,6,7].

Another plausible theory is that lipomas arise within the frontal bone. Bone marrow fat cells hyperplasia in trabecular bone is the origin of intraosseous lipomas as a result of an increase in intramedullary fat cells, which increases the pressure inside the bone cavity and causes the medullary cavity to enlarge; common changes are necrosis, calcification, and ossification and occur in the center of tumor [8,9]. These features were not found in our case.

Almost 4% of all soft tissue tumors in children are lipomas, which are far less prevalent [10]. They occur more often after puberty [10]. It is unclear how common deep lipomas are in children. Most published reports of deep forehead lipomas occurred in adults [11,12]. Lipomas often show asymptomatic, soft, flexible subcutaneous nodules, while deep forehead lipomas are solid and stiff and may feel bound down. Although an accelerated growth phase has been documented, they often appear randomly and grow slowly [2].

Differential diagnoses in children are dermoid cysts, epidermoid cysts, hemangioma, and lymphangioma [13]. A positive diagnosis can be made through imaging tests such as medical ultrasonography, histology, or clinical [14]. In the pediatric population, an MRI and a head CT scan are recommended for diagnosis [5].

Ultrasonography is frequently utilized but may not be as precise for determining the depth of the lipoma [15]. On computed tomography (CT) or MR imaging, the appearance of lipomas and their variants is more distinctive, allowing for a more precise diagnosis. When characterizing lipomas of the head and neck, MR imaging is frequently regarded as the technique of choice since it offers an improved soft tissue definition. Preoperative ultrasound was 100% precise in establishing the diagnosis in 42 patients with forehead lipomas but less accurate in defining the depth [16]. It was also demonstrated that MR imaging had 100% sensitivity and 83% specificity in diagnosing well-differentiated liposarcoma and was 100% specific in diagnosing simple lipoma [17]. Our patient underwent a CT scan of the head, which provided enough information to carry on with the surgical procedure. Invasion of the frontal bone remained exceptional and was seen on our patient's CT.

Deep lipomas that require treatment are surgically removed, and plastic surgery is mandatory. The goal is a complete removal of the lesion to minimize the risk of recurrence while preserving the surrounding normal tissue and reconstructing the frontal bone defect [1-7,10-15].

Large tumors in the frontal region of the skull can occasionally grow to the orbital bone, inflating and squeezing the eyeball's inner wall and impairing vision and eye movements [18]. Often, the lesion is removed surgically by curettage or resection [19]. Artificial materials, such as titanium plate support or 3D printing, can be implanted for sculpting severe bone abnormalities associated with lipomas. [8]

When they are heavily encapsulated, and there is little chance of recurrence or harm to nearby structures, conservative surgical excision is the most frequently advised approach [2,6,7,10-15,20]. When a lesion is not encapsulated, the recurrence risks are higher because dissection is more difficult. This difficulty could potentially harm the facial nerve, compromising esthetics functions [21]. As a result, surgical techniques that are esthetic are frequently used by surgeons to remove facial lipomas.

In our case, evolution, size, and symptoms were noticed and indicated surgical management. Multi-disciplinary planification was required, including teams of neurosurgeons, maxillo-facial surgeons, and pediatric physicians. The skin incision results were satisfactory in our patient. However, we could not identify the capsule limits, but the macroscopic removal was deemed complete. The underlying frontal bone defect presented a hole corresponding to the lipoma size. The Dura mater was intact, and the reconstruction was made possible by using a parietal flap.

Other reported treatment options included in situ administration of steroids or deoxycholate, intralesional steroids including isoproterenol, and liposuction. Children's deep forehead lipomas may not change over time or gradually become less noticeable. Short-term follow-up was marked in our patient with a good esthetic outcome of the reconstruction and good scaring.

Unfortunately, midterm follow-up revealed a right parietal extradural hematoma, possibly explained by slow post-operative bleeding from the dura mater or bone. This postoperative complication occurred in our patient despite following the appropriate recommendation and was managed with symptomatic treatment and monitoring, resulting in a regression of the symptoms and hematoma.

## Conclusions

Although deep forehead lipomas are uncommon, physicians must be familiar with them. The lesions may fade with time, but they may also be accompanied by esthetic problems and functional complications such as vision impairment and pain. Generally, they are sporadic. Imaging should be seriously considered, and MR imaging is thought to be the best modality due to its high specificity and precision in assessing the lesion and its surrounding. To evaluate the condition of the bone and the degree of invasion, a CT scan is still required.

Surgical treatment is recommended if the lesion is evolving or symptomatic. Reconstruction of the bone and skin should be done in an esthetic manner, and follow-up should be carried out regularly to depict recurrences and complications.
